# Soft transparent graphene contact lens electrodes for conformal full-cornea recording of electroretinogram

**DOI:** 10.1038/s41467-018-04781-w

**Published:** 2018-06-13

**Authors:** Rongkang Yin, Zheng Xu, Ming Mei, Zhaolong Chen, Kai Wang, Yanlin Liu, Tao Tang, Manish Kr. Priydarshi, Xuejuan Meng, Siyuan Zhao, Bing Deng, Hailin Peng, Zhongfan Liu, Xiaojie Duan

**Affiliations:** 1 Department of Biomedical Engineering, College of Engineering, Peking University, Beijing, 100871 China; 2 Peking-Tsinghua Center for Life Sciences, Peking University, Beijing, 100871 China; 3 Academy for Advanced Interdisciplinary Studies, Peking University, Beijing, 100871 China; 4 Center for Nanochemistry, Beijing Science and Engineering Center for Nanocarbons, Peking University, Beijing, 100871 China; 5 WuXi App Tec (Suzhou) Co., Ltd, Suzhou, 215104 China; 6 College of Chemistry and Molecular Engineering, Peking University, Beijing, 100871 China; 7 Department of Ophthalmology, Peking University People’s Hospital, Beijing, 100044 China; 8 College of Optometry, Peking University Health Science Center, Beijing, 100044 China

## Abstract

Visual electrophysiology measurements are important for ophthalmic diagnostic testing. Electrodes with combined optical transparency and softness are highly desirable, and sometimes indispensable for many ocular electrophysiology measurements. Here we report the fabrication of soft graphene contact lens electrodes (GRACEs) with broad-spectrum optical transparency, and their application in conformal, full-cornea recording of electroretinography (ERG) from cynomolgus monkeys. The GRACEs give higher signal amplitude than conventional ERG electrodes in recordings of various full-field ERG responses. High-quality topographic mapping of multifocal ERG under simultaneous fundus monitoring is realized. A conformal and tight interface between the GRACEs and cornea is revealed. Neither corneal irritation nor abnormal behavior of the animals is observed after ERG measurements with GRACEs. Furthermore, spatially resolved ERG recordings on rabbits with graphene multi-electrode array reveal a stronger signal at the central cornea than the periphery. These results demonstrate the unique capabilities of the graphene-based electrodes for in vivo visual electrophysiology studies.

## Abstract (web summary)

The electrical response of the eye to optical stimulus is important in disease diagnosis but current electrodes used have limitations. Here, the authors report on the development of soft transparent graphene-based contact lens electrodes for electroretinogram recording and test the device in vivo.

## Introduction

The electroretinogram (ERG) measures the electrical potential changes at the corneal surface generated by various neuronal and non-neuronal cells in the retina in response to a light stimulus. It is commonly used in ophthalmic diagnostic testing to assess the functional integrity of the retina^1^. ERG measurements are usually performed by using a recording electrode, which is positioned in contact with the cornea or bulbar conjunctiva, in combination with a reference and a ground electrode. Corneal electrodes normally consist of a contact lens with a metal conductor around the edge (to avoid blocking the light), sometimes with an additional blepharostat implemented to ensure strict corneal contact. Due to the corneal contact, corneal electrodes typically enable measurements with relatively higher signal amplitude than conjunctival electrodes^2–4^. However, the measurements are associated with discomfort because of the direct contact of a stiff contact lens with the soft and sensitive ocular structures. In addition, contact lens electrodes tend to alter the eye’s refraction, so they are not inherently well-suited for pattern ERGs (pERG) or multifocal ERGs (mfERG), both of which require sensitivity to the geographic distribution of the stimulus^5–8^. Conjunctival electrodes, placed in contact with the bulbar conjunctiva, are made of metal conductors shaped as either loops, hooks, or wires. They do not interfere with central vision and are associated with much reduced discomfort but at the cost of reduced signal amplitudes. They also promote greater eye movement, which compromises the stability and reproducibility of the ERG signals^3,4^.

Electrodes with combined softness and optical transparency could provide a superior solution for ERG measurements. First, like other electrophysiological measurements, the conformal interfacing between the soft electrodes and the curvilinear surface of cornea helps to maintain efficient and stable electrical and mechanical contacts essential for high signal amplitude and stability^9,10^. Soft electrodes could offer enhanced comfort because of reduced disturbance to the sensitive ocular structures. Besides, the conformal contact between soft electrodes and eyes could avoid the formation of thick inhomogeneous tear film or air gaps, important for preservation of eye’s refraction. Second, the use of a transparent conductor allows for full-cornea coverage of the recording element, which collects the ERG response from the whole corneal surface, rather than only from the periphery, as is the case in the traditional design using an opaque conductor. Besides, multiple soft transparent electrodes can be placed on different locations of the corneal surface without reducing the illuminance or affecting the uniformity of the full-field illumination, thus enabling the study of spatial differences of the ERG response across the cornea, which is important for correlating the spatial distribution of corneal potentials with that of retinal activity, and detecting local retinal dysfunction under a single full-field stimulus. Despite these potential advantages, using electrodes with combined softness and optical transparency for in vivo visual electrophysiology has not yet been reported.

Due to its broadband optical transparency and high electrical conductivity, graphene has been utilized in transparent neural electrodes for simultaneous electrophysiology, in vivo imaging, and optogenetic experiments^11,12^. By transferring graphene grown on flat copper foil onto wearable soft contact lenses, transparent eye interfacing devices have been developed which has been demonstrated in a variety of application including displays^13^, electromagnetic interference shielding and dehydration protection^14^, and glucose level and intraocular pressure sensing^15,16^.

Here we set out to develop a soft, transparent graphene contact lens electrode (GRACE) and use it for ERG measurements from cynomolgus monkeys and rabbits. The GRACE devices form much conformal and tighter interface with the cornea than stiff contact lens electrodes and record ERG from the whole cornea. Compared to conventional ERG electrodes, they exhibit combined advantages of high signal amplitude and stability, and capability of high-quality mfERG mapping. Furthermore, a stronger ERG signal at the center than the periphery of the cornea is observed on rabbits by spatially resolved ERG recording with soft transparent graphene multi-electrode array. We expect that with the combined softness and optical transparency, the graphene electrode technology would ultimately play a critical role in in vivo visual electrophysiology studies.

## Results

### Device preparation and characterization

**Fig. 1:**
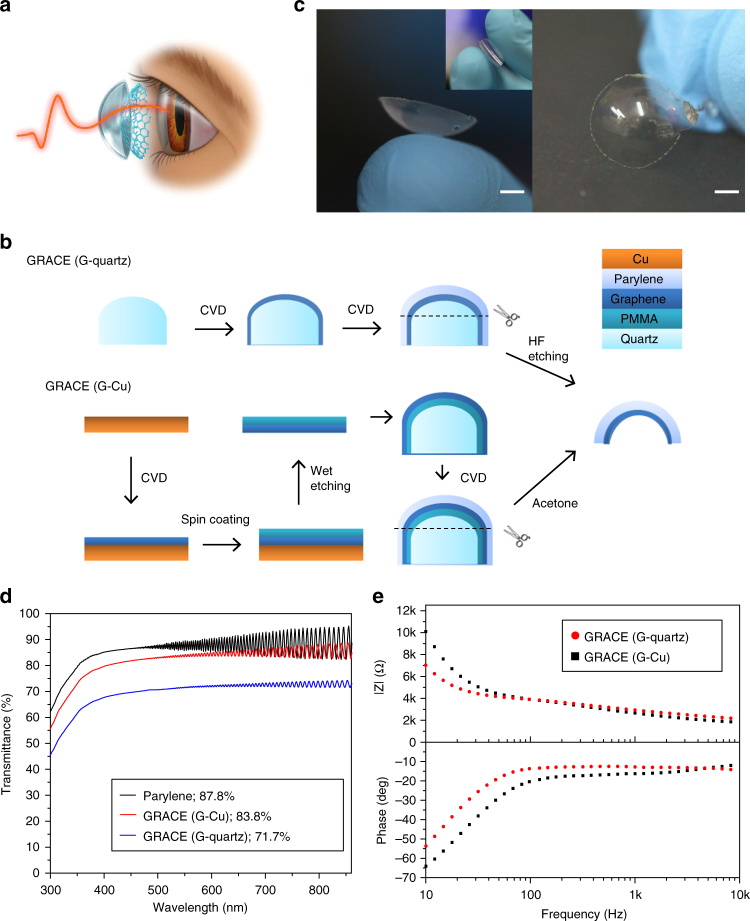
GRACE device fabrication and characterization. **a** Schematic drawing of ERG recording with the GRACE device. **b** Schematic illustration of GRACE fabrication with G-quartz and G-Cu. **c** Photographs of a GRACE device made from G-quartz. Scale bar, 3 mm. Image in the inset demonstrates the high softness of the GRACE device. **d** Optical transmittance of the bare Parylene-C, and GRACE devices made from G-quartz and G-Cu, all with Parylene thickness of 25 μm. The transmittance at 550 nm wavelength is shown in the inset. **e** Magnitude and phase of electrochemical impedance of GRACE devices measured in 1× PBS (pH 7.4)

The GRACE device consists of two layers: a soft and transparent substrate with the shape of an ocular contact lens, and a conducting graphene layer on the concave side of the substrate (Fig. 1a). A thin metal wire was connected to the graphene either on the concave or convex side to interface with the data acquisition system. While there are many options for the soft substrate, here we chose Parylene-C film, which is widely used in neural interfacing devices as substrate or insulating material^9,17^. A thickness of 5–25 μm was used to make the GRACE devices lightweight and pliable. For the graphene layer, we used that directly grown on the curved surface of a lens-shaped quartz mold, which is designed to have same curvature and size as the cornea of the recording subject, using a low-pressure chemical vapor deposition (LPCVD) process^18–21^. A simplified schematic of the fabrication process is shown in Fig. 1b. Parylene-C film was first deposited onto the as-grown graphene/quartz lens complex^22^. The Parylene/graphene stack was then released by etching the quartz in buffered hydrofluoric acid (HF). Connecting a thin metal wire to the graphene with subsequent insulation of the connection site completed the GRACE fabrication (see Methods section for details). By simply changing the curvature of the quartz mold substrate, the resulting GRACE devices can be shaped to fit eyes of different animals and animals of different ages.

The use of a curved graphene film grown directly on a lens-shaped quartz mold ensured that the devices were free of wrinkles and folds, which was important for maintaining optical homogeneity (Fig. 1c). A plot of the light transmittance vs. wavelength for bare Parylene-C showed the usual sinusoidal form^23^ and the transmittance of the GRACE device was >70% in the visible to near-infrared range (Fig. 1d), thus enabling efficient full-cornea recording of full-field ERG (ffERG) and mapping of mfERG signals. From these curves, we calculated that >80% of the light impinging through the substrate was transmitted by the graphene in the visible to near-infrared range. Measurements of the corresponding electrochemical impedance spectra (Fig. 1e) gave impedance value of 4.85 ± 0.36 kΩ and phase angle of −19.17 ± 3.32 at 100 Hz (mean ± s.d., *n* = 10 electrodes). This impedance value meets the requirement of most commercial ERG recording amplifiers.

Alternatively, GRACE devices can be fabricated from graphene grown on the surface of a flat copper foil (G-Cu) using LPCVD (Fig. 1b). The as-grown graphene was transferred onto the lens-shaped quartz mold, with the transfer-assisting PMMA layer placed in between the graphene and the quartz. After depositing Parylene-C film onto the graphene/PMMA/quartz complex, the Parylene/graphene stack was released from the quartz by dissolving the PMMA in acetone. Compared to the graphene grown on quartz (G-quartz), G-Cu showed high electrical conductivity (sheet resistance ~850 Ω/sq vs. ~1520 Ω/sq). The GRACE devices made from G-Cu also showed higher optical transparency (83.8% vs. 71.7% at 550 nm) and lower electrochemical impedance (3.39 ± 0.30 kΩ vs. 4.85 ± 0.36 kΩ at 100 Hz, mean ± s.d., *n* = 10 for each type of electrode). The G-quartz was often multilayered and associated with a smaller domain size and a higher density of defects compared to the G-Cu, as indicated by the Raman spectra (Supplementary Fig. 1)^19,20,24^. This explains the higher graphene film sheet resistance, lower optical transparency, and higher electrochemical impedance of the G-quartz devices. However, the transfer of graphene film from a flat to a curved surface resulted in graphene folding and wrinkles with PMMA residues at some locations, which makes the optical transmission not uniform across the surface of the G-Cu GRACE devices. The uniform graphene thickness and associated optical transparency and electrical conductivity across the entire contact lens electrode surface for G-quartz GRACE devices is advantageous over G-Cu GRACE devices and previously reported graphene-based eye interfacing devices. We found that the G-Cu GRACE devices can record high-quality ffERG signals but the optical inhomogeneity across the electrode surface makes them unsuitable for topological mapping of the mfERG response. All the data presented in this paper were recorded with GRACE devices from G-quartz.

### Full-field ERG recording

**Fig. 2:**
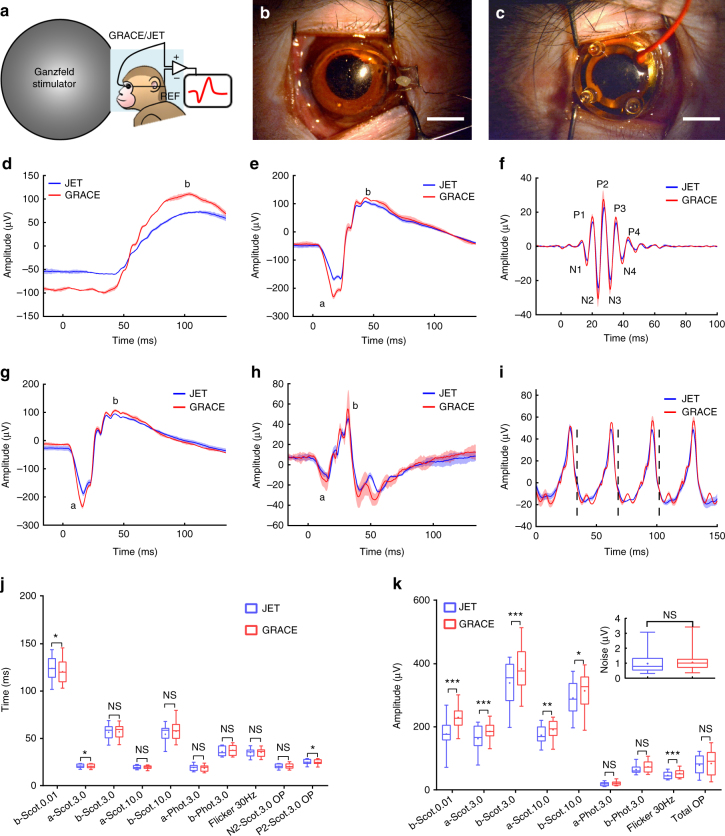
Full-field ERG recording. **a** Schematic of ffERG recording with ganzfeld stimulation on cynomolgus monkeys. **b**, **c** Photograph of a GRACE device and Jet electrode applied to an eye of a cynomolgus monkey, respectively. Scale bar, 5 mm. **d**–**i** Representative ffERG signals recorded with a GRACE device (red) and a Jet electrode (blue) from the same eye of a cynomolgus monkey, following the guidelines set by the ISCEV. The solid lines show the average signal and the shaded regions show standard deviation. *n* = 3 from same eye. **d**, **e** Scotopic (dark-adapted) ERG responses under 0.01 and 3.0 cd s m^−2^. **f** Scotopic oscillatory potentials (OPs) recorded under 3.0 cd s m^−2^. **g** Scotopic ERG responses under 10.0 cd s m^−2^. **h** Photopic (light-adapted) ERG responses under 3.0 cd s m^−2^. **i** Photopic 30 Hz flicker ERG responses under 3.0 cd s m^−2^. Different categories are presented here according to the order of recording. ‘a’ and ‘b’ mark the cornea-negative a-wave and the cornea-positive b-wave, and N1, N2, N3, P1, P2, P3…label the wavelets in oscillatory potentials. The dashed lines in **i** mark the midpoints of the stimulus flashes. Note that the a-wave for scotopic ERG under 0.01 cd s m^−2^ is absent because of the weak stimulus. **j**, **k** Summary of the implicit times and amplitudes of various ERG responses recorded by the GRACE and Jet electrodes on cynomolgus monkeys. Scot. and Phot. are the abbreviations for scotopic and photopic, respectively. The number marks the stimulus intensity. The measurements of implicit times and amplitudes for characteristic waves in various ERG responses can be found in Methods section and Supplementary Figure 2. Inset of **k**, RMS noise level comparison between GRACE and Jet electrode recordings. The bottom and top of the box are the first and third quartiles, the band and dot inside the box is the second quartile (the median) and mean value, respectively, the ends of the whiskers represent the minimum and maximum of all of the data. ***p ≤ 0.001, **p ≤ 0.01, *p ≤ 0.05, NS, *p* > 0.05; *n* = 30 from 10 eyes, Bonferroni correction for *p*-value, Wilcoxon signed-rank test analysis

The ffERG measures the ERG signal originating from the entire retina with a Ganzfeld flash stimulation; it serves as an important diagnostic clinical tool in ophthalmology for evaluating the integrity of the retina^1^. We tested the ffERG recording capability of the GRACE devices on the eyes of cynomolgus monkeys (Fig. 2a, b), an important non-human primate model for assessing ocular defects in humans. The commercially available ERG-Jet electrode, with an impedance of ~670 Ω at 100 Hz and made of plastic speculum structures with a gold-plated circumference, was used to record the ffERG signal for comparison (Fig. 2c). It can be seen that the thin, transparent GRACE device closely conformed to the front surface of the eye owing to its compliant nature. This pliable and conforming interface was important to maintain a stable, efficient, and minimally invasive electrical contact with the eye necessary for ERG recording of high signal amplitude and stability with least disturbance.

Recordings were carried out using ganzfeld full-field stimulation on a commercial RETImap system (Roland Consult, Germany), following the guidelines set by the International Society for Clinical Electrophysiology of Vision (ISCEV)^25^. Representative ffERG responses recorded by the GRACE and Jet electrodes from the same eye of a cynomolgus monkey are shown in Fig. 2d–i. Scotopic ERG responses were recorded in the dark after 30 min of dark adaptation at a luminous strength of 0.01, 3.0, and 10.0 candela-seconds per square meter (cd s m^−2^, Fig. 2d, e, g). The major components of an ERG signal are the cornea-negative a-wave and the cornea-positive b-wave. The a-wave is derived from the cones and rods of the outer photoreceptor layers and the b-wave reflects post-synaptic bipolar and Muller cell activity, in turn driven by photoreceptor input^26^. Under scotopic conditions, mainly rod function is reflected under dim stimulus flashes (Fig. 2d). With the application of brighter white light stimulus (Fig. 2e, g), combined rod and cone function is measured in ERG signal along with an increase in amplitude of the a- and b-waves. Both electrodes gave high signal-to-noise ratio scotopic signals with well-defined features characteristic of standard ERGs in the cynomolgus monkeys. And importantly, the GRACE device gave a stronger signal than the Jet electrode.

By raising the high-pass filter, the slower a- and b-wave components were filtered out leaving a burst of high-frequency, low-amplitude wavelets (dark-adapted oscillatory potentials; Fig. 2f). Oscillatory potentials are thought to reflect activity initiated by amacrine cells in the inner retina^27^, which are significantly attenuated in various forms of retinal degeneration^28^. The wavelets in oscillatory potentials are normally labeled N1, P1, N2, P2, N3, P3..., with initial negative and positive deflections defined as N1 and P1, and a second negative and positive deflection as N2 and P2, etc. (Fig. 2f). Both electrodes gave prominent and easily-detectable oscillatory potentials, with larger signal amplitudes observed in the GRACE recording. We also obtained the photopic ERG (Fig. 2h) and the light-adapted 30-Hz flicker ERG (Fig. 2i), both under a luminous strength of 3.0 cd s m^−2^. These two types of response come from the cone-mediated response, because the rod system is desensitized by the bright background light (for photopic ERG) and rods cannot follow a flicker frequency >20 Hz (for light-adapted 30-Hz flicker ERG)^29^. The GRACE device gave an a-wave comparable to the Jet electrode, but a stronger b-wave and flicker waves.

Higher amplitude of ffERG recordings by the GRACE devices than the Jet electrodes was also observed in all other tested eyes, including nine eyes from cynomolgus monkeys and four eyes from albino rabbits. For each eye, ffERG recordings were performed with one kind of electrode and immediately repeated with the other one. The implicit times, which measure the intervals from the onset of the stimulus to the wave peaks, and the amplitudes of characteristic waves in various ERG responses recorded by the GRACE and Jet electrodes on cynomolgus monkey eyes are listed in Fig. 2j, k (for details of definition and measurement, see Methods section and Supplementary Fig. 2). It can be seen that the mean amplitudes from GRACE recordings are higher than those from Jet electrode recordings for all waves. For most waves, the implicit times showed no statistically significant difference between the two electrodes, while the amplitudes recorded by the GRACE devices were larger than those recorded by the Jet electrodes. Besides, the ffERG recordings from albino rabbits showed similar results of comparable implicit times and higher amplitudes for GRACE recordings than Jet electrode recordings (Supplementary Fig. 3). Our results show that GRACE devices give higher ffERG signal amplitude than the Jet electrodes despite their ~7 times higher impedance value. We believe this stronger signal arises from the combined softness and optical transparency of the GRACE devices as will be discussed later. In future higher quality graphene growth with increased electrical conductivity can lower the impedance and further increase the ERG signal amplitude. We also measured and compared the root mean square (RMS) noise from GRACE and Jet electrode recordings, as shown in inset of Fig. 2k. Both electrodes give noise level 1~2 orders of magnitude smaller than the signal amplitude of various ffERG waves and no statistically significant difference was observed between the two electrodes.

### Multifocal ERG recording

Different from ffERG, which records the corneal potential when the entire retina is photo-stimulated, the mfERG technique allows the recording of ERG responses when small retinal areas are independently stimulated^6,29,30^. Normally in mfERG, the retina is stimulated with an array of hexagonal elements. Each hexagon is an independent stimulus. During the stimulation, each hexagon goes through a same pseudo-random binary sequence of black (no flash) and white (brief flash) presentations (m-sequence), with the starting point displaced in time relative to other elements. A single continuous ERG recording is obtained. By correlating the continuous ERG signal with the sequence of on- and off-phases of each element, multiple ERG recordings reflecting the retinal response to each of the corresponding stimulated areas are extracted. Although relatively new, the mfERG technique is used widely to diagnose and study retinal diseases. Because mfERG recording requires sensitivity to the geographic distribution of the stimulus, the electrode used must have proper refraction and avoid any interference with central vision. Besides, sensitivity, comfort, and stable interfacing with the eye is more critical for electrodes used in mfERG recording because the signal amplitude is about 1/1000th that of conventional ffERG, and mfERG requires relatively longer recording periods.

**Fig. 3:**
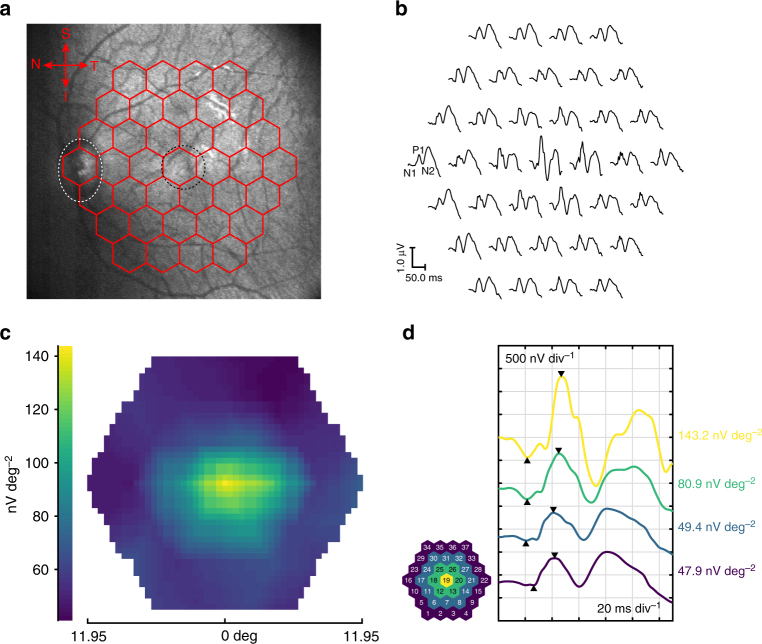
Multifocal ERG recording. **a** Infrared fundus photo of a cynomolgus monkey eye taken during mfERG recording with a GRACE device, superimposed with the stimulus array. The white dotted oval marks the position of the optic nerve head and black dotted circle marks the position of the macula. **b** Representation of trace array recorded from the cynomolgus monkey eye in **a** with GRACE. The waves of 37 focal ERG signals are topographically arranged. The principal mfERG components N1, P1, and N2 can be clearly defined in these waveforms, as labeled for one of the responses. **c** Response density plot (retinal view) on P1-wave associated with **b**. **d** The mfERG responses grouped and averaged for each of the regions marked by different colors. The values show response density of the P1 peak (as defined by the triangles on the traces) in each of the associated regions

With the GRACE devices, we successfully recorded mfERG responses from cynomolgus monkeys using the RETImap system. A representative mfERG measurement result is shown in Fig. 3. The broadband optical transparency of the GRACE devices enables simultaneous in situ infrared fundus visualization during mfERG data collection. Figure 3a shows the fundus photograph taken during the mfERG recording using a GRACE device, overlaid with the stimulus array. The optic nerve head and myelinated bands can be clearly defined in the fundus image and the presence of the GRACE device did not have adverse effects on the image quality. The stimulus pattern was positioned with the macula at the center of the stimulus array (Fig. 3a).

The trace array of the mfERG responses was topographically arranged in Fig. 3b. Because of the light levels used and the high rate of stimulation, the mfERG is mainly a response of the cone system^29^. Like the traditional photopic, or cone-driven ffERG, the mfERG shows an initial negative deflection (N1) followed by a positive peak (P1). These components bear a superficial resemblance to the a- and b- waves of the photopic flash ERG, although technically they are different and the intraretinal origins of these negative and positive waves remain obscure^29^. The GRACE device gave high-quality mfERG traces with well-defined characteristic N1 and P1 peaks. Figure 3c shows the plot of response density, which is defined as the amplitude of P1 (measured from the bottom of the N1-wave to the peak of the P1-wave) divided by the total area of the stimulus element. Clearly, highest signal amplitude is detected in the center of the stimulation area, corresponding to the macular area, which agreed well with the highest density of retinal cone here. The optic nerve head area gives weaker response; although, the difference with the area beyond the macula is not as sharp as expected from the spatial distribution of retinal cone density^31^. We think this is because of the high light scattering property of the nerve head, and the neighboring regions receiving stray light from this scattering contribute to the response^6^. This blind spot response abnormity is also documented in literature^32,33^ and referred to in the ISCEV^6^. We grouped responses in the trace array into four areas from the center to the periphery defined by different colors, as shown by the schematic in Fig. 3d. The response density is the largest in the fovea and decreases with eccentricity (Fig. 3d), agreeing well with the cone density distribution^31^. These results proved the capability of the GRACE devices to reliably map high-quality mfERG responses.

### Characterization of electrode-cornea interface

**Fig. 4:**
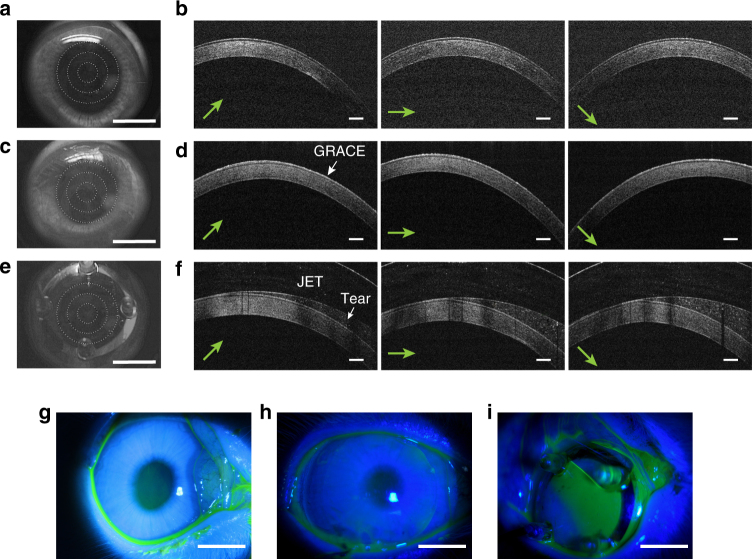
Electrode-cornea interface characterization. **a**–**f** Photograph and anterior segment OCT images of a bare rabbit eye (**a**, **b**), a rabbit eye wearing a GRACE (**c**, **d**), and a rabbit eye wearing a Jet electrode (**e**, **f**). The anterior segment OCT cross-sectional images in **b**, **d**, **f** are on corneal meridian along the directions marked by the green arrows in the lower left corners. Scale bars, 300 μm. **g**–**i** Slit lamp micrographs of a bare rabbit eye (**g**), a rabbit eye wearing a GRACE (**h**), and a rabbit eye wearing a Jet electrode (**i**), with tear film stained with sodium fluorescein. The intensity of fluorescence represents the thickness of the tear film. Scale bar in **a**, **c**, **e**, **g**–**i**, 5 mm

To shed light on the origin of the high ERG signal amplitude from GRACE recordings, we conducted anterior segment optical coherence tomography (OCT) studies of the electrode-cornea interfaces on albino rabbit eyes. As shown by Fig. 4a–f, the soft GRACE devices formed a much conformal and tighter interface with the cornea than the stiff Jet electrodes. There was little tear film between the GRACE devices and cornea, compared to the thick and uneven tear film between the Jet electrodes and cornea. The thick and uneven tear film between Jet electrodes and cornea was also visualized using sodium fluorescein staining and slit lamp examination (Fig. 4i), while the significantly lower fluorescence between the GRACE devices and cornea (Fig. 4h), with intensity comparable to that of naked eyes (Fig. 4g), indicates much thinner tear film. Corneal topography provides a detailed description of various curvature and shape characteristics of the cornea^34^. We found the measured radius of curvature of the GRACE devices worn on rabbit eyes showed no obvious difference with that of naked eyes (Supplementary Fig. 4), indicating conformal interface between the GRACE devices and cornea. The conformal and tight interface between the soft GRACE devices and cornea will be important for high ERG signal amplitude and preservation of visual acuity, as will be discussed later.

### Multi-electrode ERG recording

By applying multiple microelectrodes at different locations on cornea, localized ERG responses can be mapped across the corneal surface under single full-field light stimulus. This multi-electrode ERG (meERG) recording enables elucidating the relationship between the spatial distribution of corneal potentials and that of retinal activity, and validating bioelectric models of the eye for electrophysiological functional imaging of the retina^35,36^. Besides, retinal lesions are known to cause change of the local corneal potentials which makes the meERG recording a potential diagnostic tool for detecting local areas of retinal dysfunction under single full-field stimulus^35,37,38^. Ideally, the electrodes used in meERG should be optically transparent so that they will not reduce illuminance on retina or affect the uniformity of the full-field illumination across the retina. This is especially important for high spatial resolution mapping of corneal potential with high-density electrode arrays. And a tight interface between the electrodes and cornea with minimized tear film is desired to avoid shunting of potential differences by the tear film. Here we show that these challenges can be met with the soft graphene microelectrode array by simultaneous meERG recording from the cornea of ophthalmologically normal albino rabbits.

**Fig. 5:**
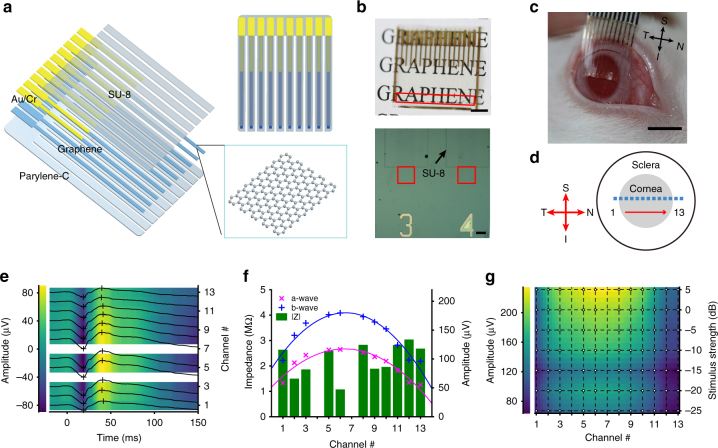
Multi-electrode ERG recording with soft, transparent graphene electrode array. **a** Diagram of graphene multi-electrode array construction showing the layered structures. **b** Top, a soft, transparent graphene electrode array positioned over a printed paper to show its optical transparency. Scale bar, 5 mm. The recording sites, arranged in a linear pattern, are located in the region marked by the red box. Under each recording site, there is a channel number patterned with Au which is optically opaque. Bottom, optical microscopy image showing some of the graphene electrode sites and traces. The red box marks the graphene recording sites. The black arrow points to the patterned SU-8 insulation layer on one electrode. Scale bar, 150 μm. **c** A stripped graphene electrode array positioned over a dilated rabbit eye. Scale bar, 5 mm. **d** A schematic drawing showing the positions of the recording channels (marked by the squares) on a rabbit eye. Channel 1 to 13 was evenly distributed over equator of the cornea from temporal to nasal periphery. **e** A representative set of the multi-electrode scotopic ERG response waveforms. Stimulus strength, 0.3 cd s m^−2^. The placement of the graphene electrode array is shown in **d**. The crosses mark the positions of the a and b- waves. Channels 4 and 7 have abnormally high impedance and are considered non-functional. **f** Plots of the electrode impedance values |Z| at 100 Hz, a- and b-wave amplitudes of the ERG signals recorded from different channels associated with **e**. The lines show the quadratic curve fitting of the a- and b-wave amplitudes. **g** Spatial profile of b-wave amplitudes under different stimulation strength. 0 dB corresponds to 3.0 cd s m^−2^. The dots in the overlaid grid mark the positions with actual experimental data

The soft, transparent graphene microelectrode array was made using the conventional microfabrication process. The array contained graphene recording microelectrodes with recording site size of 300 × 300 μm^2^, with spacing of 1 mm arranged in a line on Parylene-C substrate. SU-8 was used as the insulation layer for the connection line. The connection pads and following portions of the connection lines were patterned with gold, while the recording sites and portions of the connection lines to be in contact with the eye were left with only graphene to maintain optical transparency for photo-stimulation of the retina (Fig. 5a, b). To form a robust interface with the curved surface of the rabbit eyes, we cut the electrode array into separate strips, each of which contained one electrode. The effective bending stiffness per width of the graphene microelectrodes are 1.67 × 10^2^ nN m and 8.42 × 10^3^ nN m for 5 μm and 25 μm thick Parylene-C substrates, respectively (Supplementary Methods), which is two to three orders of magnitude smaller than the values reported for previous implantable electronics, such as silicon (4.6 × 10^5^ nN m) and carbon fiber (3.9 × 10^4^ nN m) electrodes^39^. The linear recording arrays were aligned over the equator of the rabbit eyes, spanning from the nasal to the temporal periphery of cornea, and the electrodes were evenly distributed along the nasal-temporal axis (Fig. 5c, d). We recorded scotopic ERG responses in response to a single ganzfeld flash of different stimulus strength simultaneously from all channels using an Intan system.

In a representative recording, the electrodes were positioned as Fig. 5d shows and the meERG response waveforms from different channels under stimulation intensity of 0.3 cd s m^−2^ were plotted in Fig. 5e. Each channel gave a typical scotopic ERG signal, with characteristic a- and b- waves, similar to conventional single-electrode ffERG recording. The a- and b- wave amplitudes were clearly higher at the central cornea region than the periphery and showed a descending trend along with the increase of distance from the cornea center (Fig. 5e, f). Besides, the position-dependence of the a- and b- wave amplitudes showed no correlation with the distribution of electrodes impedance (Fig. 5f). This precludes the impedance difference across different channels as the origin for the spatial differences in a- and b-wave amplitudes. The b-wave amplitude distribution across different channels under various stimulus strength is plotted in Fig. 5g (corresponding ERG traces under each stimulus strength can be found in Supplementary Fig. 5). Same spatial profile of ERG response with amplitude decreasing along with the increase of distance from the cornea center was also observed from meERG recording on other rabbit eyes (*n* = 12) (Supplementary Fig. 6). We suspect that this variation reflects the intrinsic spatial differences in ERG potential across the cornea, arising from regional differences in the retinal response to the stimulus, the inhomogeneous distribution in conductivity^36,40^, and/or the anatomy of the ocular tissues. Further work is needed to optimize the spatially resolved meERG recordings and elucidate the origin of the ERG potential distribution across the cornea. The demonstration here indicates that the soft, transparent graphene electrode array provides an effective platform with which to study the topographical distribution of corneal ERG potentials.

## Discussion

The choice of an appropriate recording electrode is of fundamental importance for ERG tests. Signal strength and stability, preservation of ocular refraction, and comfort are important factors to be considered when choosing an electrode for ERG recording in patients^25,29^. The ERG-Jet electrode, extensively used for clinical ffERG recording, is reported to give the highest signal amplitude among commercially available ERG electrodes^3,4^. Here, we found that the GRACE devices record stronger signals than Jet electrodes; although, the impedance of the former is ~7 times higher than the latter. We measured the optical transparency of the central plastic opening of the Jet electrodes (Supplementary Fig. 7) and found that it has optical transmission in the visible to near-infrared range comparable with the GRACE devices. This indicated that there was no difference between these electrodes in the stimulus intensity received by the eyes. We suggest that the softness and full-cornea recording capability of the GRACE devices account for their high signal amplitude. First, as indicated by the anterior segment OCT and tear film fluorescein staining results, the soft, lightweight GRACE devices conformably adhere to the curvilinear surface of the eyes, forming a tighter interface with much thinner tear film than the stiff and bulky Jet electrodes. This results in a higher sealing resistance which is known to give a higher signal amplitude^9^. Second, the GRACE devices recorded ERG from the entire corneal surface, enabled by their optical transparency. The additional contribution of ERG signals from the central cornea could increase the signal amplitude recorded by the GRACE devices. The meERG recording on rabbits observed a stronger ERG signal at the center than the periphery, which supports the above speculation. Furthermore, like other corneal contact lens ERG electrodes, the GRACE recordings were stable as indicated by the small deviation (comparable with that from Jet electrode recordings) of multiple ffERG measurements (shaded region in Fig. 2d–i). Electrode/cornea interface instability associated with eye movements and blinks is the main cause of high signal variance^25^, which often happens for conjunctival electrodes, such as DTL electrodes. We videotaped rabbit eyes wearing the GRACE devices and found that the GRACE devices can remain on the cornea with no noticeable movements under eye blinks (Supplementary Movie 1), suggesting a stable interfacing under eye motions, as expected for corneal contact lens electrodes.

Stringent requirements for visual acuity need to be met during mfERG recording. Many corneal contact lens electrodes, such as the Jet electrodes, are not normally used in mfERG recording because they tend to alter the eye’s refraction^2,41^. The existence of thick and uneven tear film in the gap between the stiff contact lens electrodes and cornea can induce a refractive error. If there is air trapped in this gap, visual acuity could be further affected. Conjunctival electrodes such as DTL electrodes are preferable for mfERG recording, but they suffer from low signal sensitivity and stability^8,29^. Here we showed that the GRACE devices, with high signal amplitude and stability, are well-suited for mfERG recording. The much conformal and tighter interface between GRACE devices and cornea avoids the formation of thick, uneven liquid or air gap between electrodes and cornea, which we believe could ensure a good preservation of the eye’s refraction. We found that it is hard to accurately measure the refractive error from animal eyes due to the challenge of controlling the ocular position and taking up fixation with the fovea. In future the direct measurement of the refractive error from human eyes wearing GRACE devices will be helpful to evaluate the effect of the GRACE on visual acuity. Patient comfort and minimal ocular irritation is another important consideration for ERG recording. We performed corneal fluorescein staining on rabbit eyes after 30 min GRACE devices wearing and no obvious staining was found on the surface of the cornea (Supplementary Fig. 8), indicating negligible corneal irritation from GRACE wearing for ERG measurement relevant time. Besides, we found that when the rabbits wear GRACEs, their eyes can blink normally (Supplementary Movie 1) and no abnormal behavior such as rubbing at eyes happens (Supplementary Movie 2). We believe these results suggest little disturbance of graphene electrodes wearing on rabbits. For future development, the use of hydrogel-type soft contact lenses to replace Parylene as the substrate could further enhance the comfort.

Our results show that graphene multi-electrode array can record well-defined spatially resolved ERG responses. Earlier efforts used either two pairs of differential scleral electrodes or a single electrode moved from location to location on the surface of rabbit or dog eyes to perform meERG recording^37,42^. A contact glass fitted with 9 electrodes placed along the same meridian was used to record local ERG responses from humans^43^. By utilizing a poly (methyl methacrylate) (PMMA) contact lens with an array of through-holes which are connected to platinum electrodes on the outer surface of the contact lens, recent work obtained two-dimensional (2D) corneal potential maps in both healthy eyes and eyes with experimental retinal lesions from rats^35,38^. We note that the ERG amplitude difference between the central and peripheral cornea observed here is larger than what were reported previously^35,37,38,42,43^. It is known that the presence of the high-conductance tear film between the electrodes and cornea can shunt the ERG potential differences over the corneal surface. The soft graphene electrodes can form tight interface with the cornea with much thinner tear film compared to stiff electrodes, this can be an important factor contributing to the large ERG potential difference. In addition, the more homogeneous retinal illumination enabled by transparent graphene electrode arrays compared to opaque metal-based electrodes used in previous studies^35,38,43^ may also play a role in yielding the different spatial profile of ERG potential. For future work, the development of high-density 2D graphene multi-electrode array which can robustly and reliably interface with the cornea will enable a full corneal potential mapping, providing means for functional retinal activity imaging. This will not only be helpful to guide the design of ERG electrodes for maximum signal strength, but also can act as a diagnostic tool for detecting local areas of retinal dysfunction under a single full-field stimulus.

The present study demonstrates the application of soft transparent electrodes in in vivo visual electrophysiology measurements. With combined softness and optical transparency, the graphene electrodes showed capability for high-efficacy measurements of various kinds of ERG signals, including ffERG, mfERG, and meERG, with negligible corneal irritation. This unique multifunction is not achievable with other available ERG electrodes. We anticipate that the graphene electrode technology introduced in this report will offer unique research capabilities in ocular electrophysiology studies.

## Methods

### Graphene growth

The G-Cu was grown on copper foil 25 μm thick (Alfa-Aesar #46365) in a home-made low-pressure chemical vapor deposition (LPCVD) system after electrochemical polishing of the copper foil. Growth was carried out under a flow of H_2_ and CH_4_ (50:1 in volume) at 55 Pa and 1010 °C for 45 min. For the growth of G-quartz, the lens-shaped quartz was thoroughly cleaned with deionized water, acetone, and ethanol before being loaded into a horizontal quartz tube placed inside a three-zone high-temperature furnace. The chemical vapor deposition system was flushed with 500 sccm Ar to remove air and the furnace was then heated to 1030 °C and stabilized for ~15 min under mixed carrier gas (30 sccm H_2_ and 150 sccm Ar). Then CH_4_ (8 sccm) was added to the carrier gas for graphene growth for 2–4 h. The graphene thickness was controlled by adjusting the growth time. Raman spectroscopy was carried out on the as-grown graphene on quartz, while for G-Cu, the graphene was transferred to a 300 nm SiO_2_/Si substrate for Raman characterization (Jobin-Yvon Horiba LabRAM HR-800, 514 nm, ×100 objective, France).

### GRACE fabrication and characterization

For GRACE fabrication with G-quartz, Parylene-C film 5–25 μm thick was first deposited onto the as-grown graphene/quartz lens complex, in a home-made low-pressure coating system. Briefly, Parylene-C dimer powder (C_16_H_14_C_l2_, J&K Scientific) was sublimed at 150 °C. After pyrolysis at 650 °C, the polymer was deposited on the graphene/quartz at room temperature. The thickness of the Parylene-C film was controlled by the amount of powder. Then an incision was made into the Parylene/graphene to remove the part on the flat side and expose the quartz for subsequent buffered hydrofluoric acid etching. An extra patch of Parylene/graphene beyond the contact lens electrode was left for metal wire connection (Fig. 1c). After etching the quartz substrate, the Parylene/graphene stack was thoroughly washed in deionized water and dried. Finally, a varnished copper wire 100 μm in diameter was connected to the graphene on the extra patch of the Parylene/graphene stack using silver paste, either on the concave or the convex side, to interface with the data acquisition system. For the convex side copper wire connection, the extra patch of Parylene/graphene was folded over onto the convex side to provide a connection site. Insulation of the connection site with epoxy finished the fabrication.

To fabricate the GRACE device with G-Cu, the as-grown graphene on copper foil was transferred to the curved surface of lens-shaped quartz through a PMMA-assisted process, with the PMMA layer placed between the graphene and the quartz. After the Parylene-C deposition (as above), the Parylene layer on the flat side and the unnecessary part of the Parylene/graphene/PMMA stack was cut away. The Parylene/graphene stack was then released from the quartz by dissolving the PMMA in acetone, thoroughly rinsed with deionized water, and dried. The subsequent copper wire connection and insulation were as above.

The optical transmittance measurements were conducted using UV-Vis spectroscopy (PerkinElmer Lambda 950 spectrophotometer). Electrochemical impedance spectroscopy measurement was performed in 1× phosphate-buffered saline (PBS) at pH 7.4 and room temperature using a CHI660e electrochemical workstation (CH Instruments, USA). A three-electrode configuration was used, with the potentials referenced to an Ag/AgCl electrode; a large surface area platinum wire served as the counter electrode; and the tested sample was the working electrode.

### Full-field ERG recording

All procedures of handling animals used in this work were approved by the Peking University Committee on the Use and Care of Animals and were performed in compliance with the ARVO Statement for the Use of Animals in Ophthalmic and Vision Research. Ophthalmologically normal female cynomolgus monkeys of 2.7–3.5 kg body weight were randomly chosen and used for measurements. The animals were first sedated by intramuscular injection of ketamine (10 mg kg^−1^), and then anesthetized by intravenous injection of pentobarbital (30 mg kg^−1^). The pupils were fully dilated with topical cyclopentolate hydrochloride 1% (Cyclogyl). The GRACE devices and commercial ERG-Jet electrodes were applied to the topically anesthetized cornea. For each animal, we applied the GRACE and Jet electrodes on two different eyes for one set of ffERG recordings, and then the two electrodes were switched to the opposite eyes for another set of ffERG recordings. This way half of the eyes were tested with GRACE first, Jet electrode secondly, and the other half were tested with Jet electrode first, GRACE secondly. This approach minimizes the potential influence of the light stimulation sequence on the comparison of the ERG responses from different electrode types, and thus yields a meaningful result. A subcutaneous platinum needle electrode placed 0.5 cm posterior to the lateral canthus over the zygomatic arch was used as reference and the ground platinum needle electrode was placed subcutaneously at the tail root. Electrode impedance was checked before recording. The ffERG testing was performed using a RETImap system (Roland Consult, Germany), following the guidelines set by the International Society for Clinical Electrophysiology of Vision (ISCEV). Full-field stimulation was achieved using a ganzfeld sphere, which presented the eye with an extensive and evenly illuminated field of view for both short flashes and steady background illumination.

Scotopic ERGs were recorded in the dark after 30 min of dark adaptation. Responses were obtained with a wide-band filter (−3 dB at 0.3 Hz and 300 Hz), stimulating with single Ganzfeld flashes (2 ms) of white light. Scotopic ERG oscillatory potentials were obtained by applying an overall band-pass filter from 75 to 300 Hz on the scotopic ERG waveforms under 3.0 cd s m^−2^. Before photopic ERG recording, 10 min light adaptation was carried out. The same single full-field flashes and filtering as scotopic ERG were used for photopic ERG recording, which was taken under luminous energy of 3.0 cd s m^−2^. A train of brief (5 ms), full-field white light flashes of 3.0 cd s m^−2^ at 30 Hz was applied for the 30 Hz flicker ERG recording. The same filtering of 0.3–300 Hz was used. Three recordings were repeated and averaged for scotopic, oscillatory potentials, and photopic signals at each intensity and eight recordings were repeated and averaged for 30 Hz flicker ERG.

### Full-field ERG signal analysis

The major components of scotopic and photopic ERG signals are the cornea-negative a-wave and the cornea-positive b-wave. Note that the a-wave for scotopic ERG under 0.01 cd s m^−2^ was absent because of the weak stimulus. As shown schematically in Supplementary Fig. 2, the amplitude of the a-wave was measured from the pre-stimulus baseline to the trough of the a-wave, while the amplitude of the b-wave was measured from the trough of the a-wave to the peak of the b-wave. The a-wave implicit time was measured from the flash onset to the trough of the a-wave, and b-wave implicit time from the flash onset to the peak of the b-wave. The overall oscillatory potential response amplitude (marked as ‘Total OP’ in Fig. 2k) is measured as the sum of P1 to P4 wave amplitudes, where the Px amplitude was measured from the trough of the Nx-wave to the following positive peak of the Px-wave. The N2 and P2 implicit times for scotopic oscillatory potentials (marked as ‘N2-Scot. 3.0 OP’ and ‘P2-Scot. 3.0 OP’ in Fig. 2j) were measured from the flash onset to the trough of the N2-wave and from the flash onset to the peak of the P2-wave, respectively. The amplitude of the 30 Hz flicker ERG was measured from averaging trough-to-peak amplitude of the first four positive waves. The peak time of the 30 Hz flicker ERG was measured from the midpoint of the stimulus flash to the following peak; the first four peaks were averaged. RMS noise was calculated from a 50 ms baseline before light stimulation. Some traces show a sign of mains frequency for both electrode recordings but it doesnot affect the RMS noise values.

Since GRACE and JET electrodes were tested on the same eyes for each stimulus condition, we regarded the amplitude, implicit time and noise level of ERG responses recorded by GRACE and JET electrodes as paired data. The Shapiro-Wilk test was used to test the normality of the sample data and residuals, and the results showed some are deviated from the normal distribution. Wilcoxon signed-rank test, a non-parametric statistical paired difference test which doesnot need assuming the population to be normally distributed, was used to compare the differences between GRACE and JET electrode recordings of various ERG responses under different stimulus conditions. Bonferroni correction was used to counteract the issue of raising type I error due to multiple tests. All tests were performed two-sided. A *P*-value of <0.05 was considered significant. All statistical analyses were performed using the SPSS version 24.0 Software (IBM, Armonk, New York). Recordings from ten monkey eyes were analyzed. Sample sizes were estimated based on previous similar studies. Animal experiments were not blinded.

### Multifocal ERG recording

General and topical anesthesia was administered as above. GRACE devices were applied to fully dilated eyes of cynomolgus monkeys, with reference and ground Pt needle electrodes applied as above. The recordings were made using the RETImap system (Roland Consult, Germany). In a typical recording, using scanning laser ophthalmoscopy (SLO), the fundus was visualized, and the stimulus pattern was consistently positioned with the macula at the center of the recording area (Fig. 3a). After each stimulus cycle, a fundus photograph from the SLO was taken to document the fundus position and ensure that there was no significant movement of the eye during the recording. An array of 37 unscaled hexagons was projected onto the retina under infrared fundus monitoring. The stimuli were generated by a projector with a refresh rate of 60 Hz. In the m-sequence, the luminance of the hexagons was either 150 cd m^−2^ (corresponding to 2.5 cd s m^−2^ in a single frame) or <1 cd m^−2^. The signals were digitalized and acquired at a sampling frequency of 1024 Hz. The responses were amplified and band-pass filtered (5–100 Hz). Six cycles were averaged for a final result. The total time for a complete mfERG measurement is ~10 min.

### Characterization of electrode-cornea interface

Ophthalmologically normal male Japanese White Rabbits of 2.0–2.5 kg body weight were randomly chosen and used. For anterior segment OCT on rabbit eyes, a RTVue XR100-2 (Optovue, Inc., Fremont, CA, USA) with a Cornea-Anterior Module (CAM) attachment was used. It has a scan area of 6 × 6 mm in the central cornea and a depth resolution of up to 5 μm. A pachymetric scan mode with eight radial scan lines was used. The light source of the system uses super luminescent diodes with a wavelength of 840 nm. All the images in this study were captured by the same experienced operator. During each scan, the operator captured each cross-sectional corneal image with the light beam at the midpoint of the cornea to ensure a centralized scan location. An artificial tear was applied as needed to prevent corneal drying.

Sodium fluorescein was instilled into the inferior sclera of rabbit eyes with normal saline moistened fluorescein sodium strips (Tianjin jingming New technological development Co.,Ltd, Tianjin, China). The fluorescein solution was then mixed in the tear film by several eye blinks. Slit-lamp microscopy (SL- 8Z, Topcon Corp., Tokyo, Japan) with a cobalt-blue filter was used to examine the eyes 3 min after the sodium fluorescein instillation. For corneal epithelium staining, the rabbit eyes were washed gently with normal saline solution after 3 min sodium fluorescein instillation in order to remove the excess sodium fluorescein in tear film. Corneal topography was conducted on rabbit eyes using Sirius corneal topographer with Scheimpflug camera and Placido-disk (CSO, Costruzione Strumenti Oftalmici srl, Florence, Italy). The instrument automatically provides mean keratometry values along the steepest and flattest meridians of the central cornea with astigmatism. SimK values (flat K, steep K, their corresponding axes, and mean SimK) were reported. All examinations in this study were performed by the same experienced operator under natural light conditions.

### Graphene microelectrode array fabrication and meERG recording

After graphene growth on copper foil, Parylene-C of 5 ~ 25 μm thick was deposited onto the as-grown graphene/copper using the process described above. Then copper foil was etched in 0.25 M Na_2_S_2_O_8_ solution to release the graphene/Parylene film. The graphene/Parylene film was transferred onto a glass slide for the following microfabrication process. Photolithography and thermal evaporation were used to define Cr/Au (8/60 nm) to form connection pads and following portions of the electrode traces. The graphene was then patterned via reactive ion etching using an oxygen plasma with photoresist as a mask, to create the graphene microelectrodes connected to each of the gold pads. Subsequently, 2 μm of SU-8 (SU-8 2002; MicroChem Corp.) was patterned by photolithography to form the encapsulation layer on the microelectrodes, with the recording sites and connection pads exposed. Finally, the array was released by peeling off from the glass substrate. A heat seal connector (HSC) was aligned and hot-bar bonded to the connection pads with low electrical resistance.

Due to the high impedance of the graphene microelectrodes, the RETImap system (Roland Consult, Germany) cannot be used for meERG measurements. Instead, we used a 32-channel Intan RHD 2132 amplifier evaluation system (Intan Technologies, USA, with input impedance of 1300 MΩ at 10 Hz and 13 MΩ at 1 kHz.) which was connected to the electrodes through the HSC and a custom electrode interface board. Rabbits were anesthetized with a mixture of 50 mg mL^−1^ ketamine and 5 mg mL^−1^ xylazine administered intraperitoneally with a dosage of 0.125 mL/100 g of body weight. The eyes were fully dilated with 0.5% phenylephrine hydrochloride and 0.5% tropicamide. The electrode arrays were cut into strips of 1 mm wide, each containing one electrode. In a typical measurement, the stripped graphene electrode array was placed on a fully dilated eye of a rabbit. The impedance of the electrodes in the array was measured before recording. Channels with impedance over 5 MΩ at 100 Hz are considered non-functional. The eye was dark-adapted for at least 20 min before multi-electrode scotopic ERG recordings. A ground Pt needle electrode was placed subcutaneously in the dorsal cervical region. The commercial Ganzfeld stimulator on the RETImap system (Roland Consult, Germany) was used to provide single full-field, evenly illuminated white light flashes (1 ms) with luminous intensity from 0.01 to 10.0 cd s m^−2^. A photo detector OPT101 (Texas Instruments, Texas, USA) was applied under the stimuli and its output was recorded by the Intan RHD 2132 amplifier evaluation system to mark the time points of the application for the light stimuli. The multi-channel signals were amplified, digitized, and acquired at a sampling frequency of 30 kHz and band-pass filtered (0.3–300 Hz). Ten flashes were repeated and averaged to obtain the final results. Twelve independent experiments were performed.

### Code availability

All code used in this work are available from the authors upon reasonable request.

### Data availability

All data supporting this work are available on reasonable request to the corresponding author.

## Electronic supplementary material

Supplementary Information

Description of Additional Supplementary Files

Supplementary Movie 1

Supplementary Movie 2
