# Supplementary material for: Soft transparent graphene contact lens electrodes for conformal full-cornea recording of electroretinogram
Source: Nat Commun. 2018 Jun 13;9:2334. doi: 10.1038/s41467-018-04781-w (PMC5998030; doi:10.1038/s41467-018-04781-w)
Supplement: Supplementary file 1 — Supplementary Information [file 41467_2018_4781_MOESM1_ESM.pdf]

Supplementary Information for

Soft transparent graphene contact lens electrodes for conformal full-cornea recording of electroretinogram

Yin et al.

Supplementary Methods

Bending stiffness calculation of the graphene microelectrodes. To calculate the effective bending stiffness per width of the graphene microelectrodes, we modeled each strip of the graphene microelectrodes as a two-layered system of Parylene-C and SU-8 because the thickness of the graphene layer is very small compared to these two layers. The bending stiffness of the electrode, E_e, can be calculated from the following equations[1]:

$$E_e = \overline{E}_P h_p \left(\frac{1}{3}h_p^2 - h_p y_0 + y_0^2\right) + \overline{E}_s h_s \left(\frac{1}{3}h_s^2 + h_s\left(h_p - y_0\right) + \left(h_p - y_0\right)^2\right) \qquad (1)$$

where h_p is the thickness of the Parylene-C film, h_s is the thickness of the SU-8, $\overline{E}_p$ represents the effective Young's modulus of Parylene-C, $\overline{E}_s$ represents the effective Young's modulus of SU-8, y_0 represents the position of the neutral mechanical plane, which is obtained as

$$y_0 = \frac{\overline{E}_P h_p^2 + \overline{E}_s h_s\left(2h_p + h_s\right)}{2\left(\overline{E}_P h_p + \overline{E}_s h_s\right)} \qquad (2)$$

where $\overline{E}_i$ represents the effective Young's modulus of each material, defined as

$$\overline{E}_i = \frac{E_i}{1 - \upsilon_i^2} \qquad (3)$$

The specific values of Young's modulus E_i and Poisson's ratio υ_i of Parylene-C and SU-8 are listed in Supplementary Table 1.

Supplementary Table 1. Young's modulus and Poisson's ratio of Parylene-C and SU-8.

Material	Young's modulus (GPa)	Poisson's ratio
Parylene-C	4.5 [2]	0.4 [3]
SU-8	3 [4]	0.26 [4]

Supplementary Figures

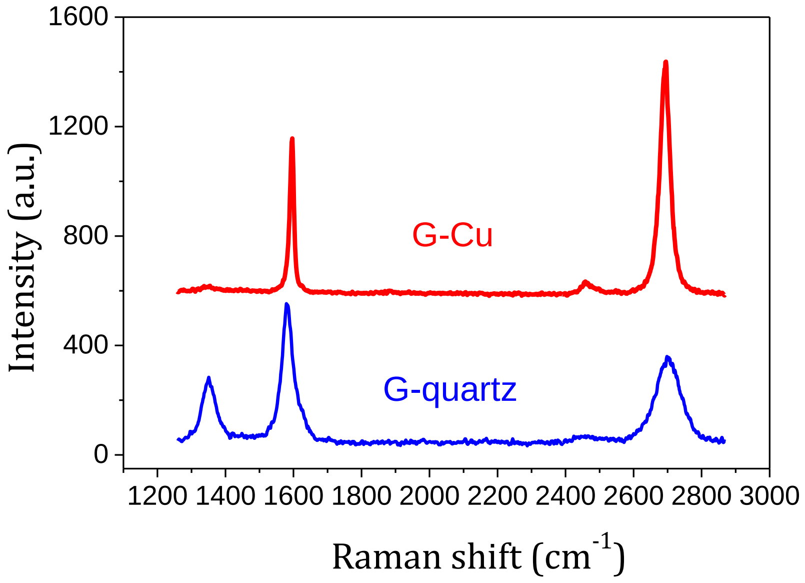

Supplementary Figure 1. Raman spectra of G-quartz and G-Cu. Raman spectroscopy was taken on the as-grown graphene on quartz, while for G-Cu, the graphene was transferred to 300 nm SiO_2/Si substrate for Raman characterization.

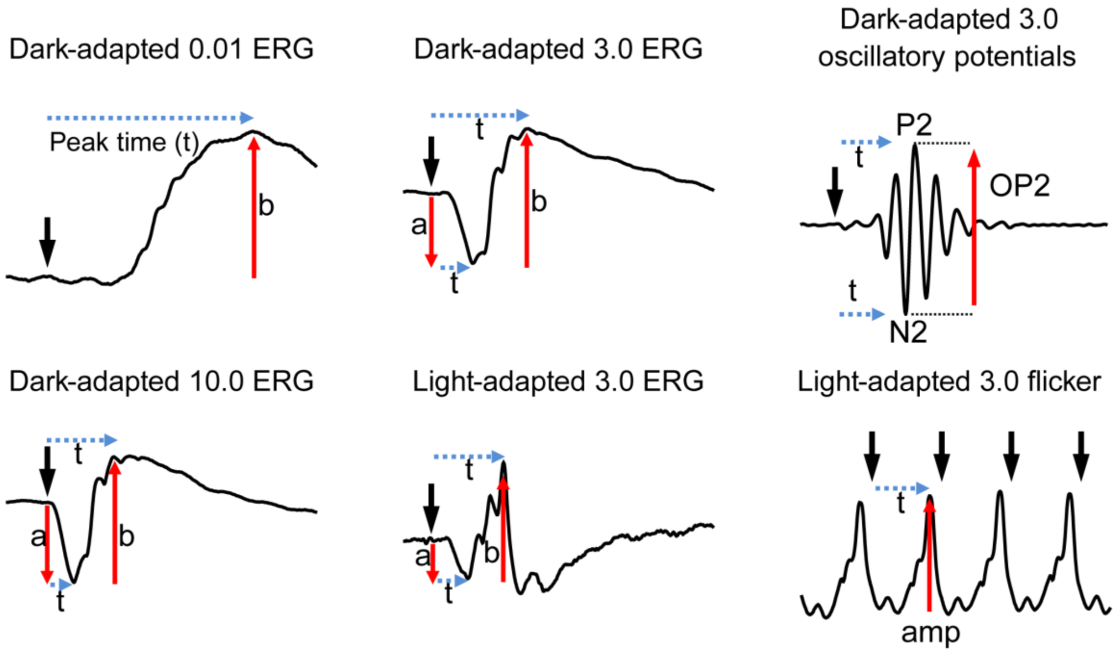

Supplementary Figure 2. Diagram of the six basic ffERG signals. Black bold arrowheads indicate the stimulus flash; Red solid arrows illustrate a-wave and b-wave amplitudes. ‘OP2’ in Dark-adapted 3.0 oscillatory potentials indicates the amplitude of the P2 wave. The ‘total OP’ shown in Fig. 2k was measured as the sum of P1 to P4 wave amplitude, where Px amplitude was measured from the trough of the Nx-wave to the following positive peak of the Px-wave. The 30 Hz flicker ERG amplitude was measured from averaging the amplitude (trough to peak, shown as ‘amp’) of the first four positive waves. Blue dotted arrows exemplify how to measure the implicit time (t, or peak time).

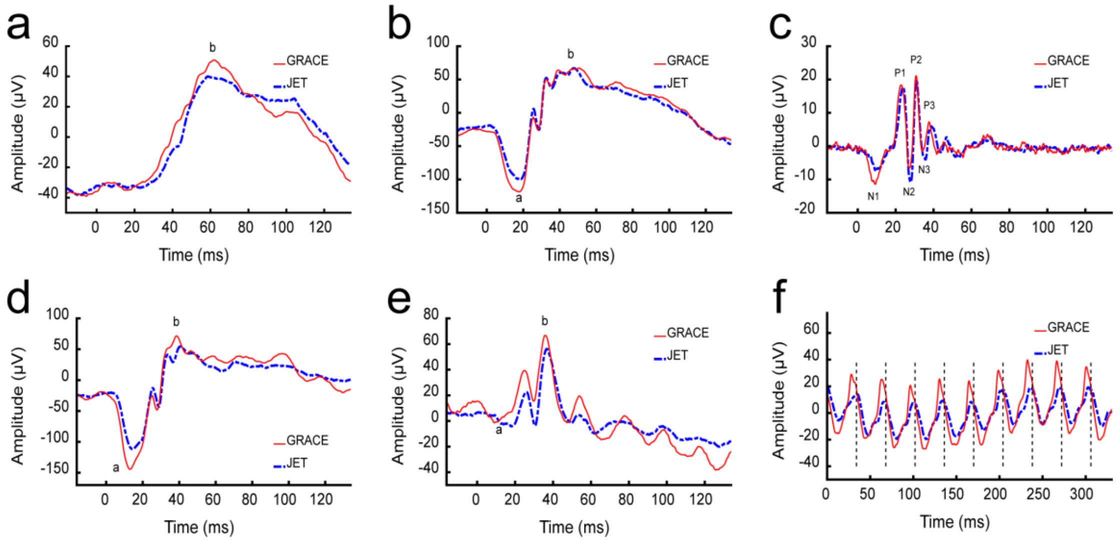

Supplementary Figure 3. Full-field ERG recording from albino rabbit using GRACE device and Jet electrode. a-f, Representative ffERG signals recorded with a GRACE device (red solid) and a Jet electrode (blue dotted) from one eye of an albino rabbit, following the guidelines set by the ISCEV. From **a** to **f**, Scotopic ERG responses under 0.01 cd s m^{-2}; Scotopic ERG responses under 3.0 cd s m^{-2}; Scotopic oscillatory potentials (OPs) recorded under 3.0 cd s m^{-2}; Scotopic ERG responses under 10.0 cd s m^{-2}; Photopic ERG responses under 3.0 cd s m^{-2}; Light-adapted 30 Hz flicker ERG responses under 3.0 cd s m^{-2}. Different categories are presented here according to the order of recording.

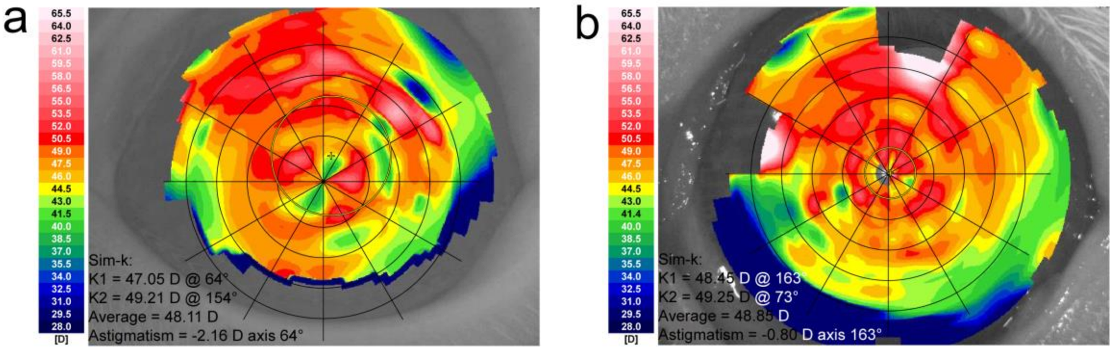

Supplementary Figure 4. A representative corneal topography tangential map recorded from the same rabbit eye without (a) and with (b) a GRACE. Simulated keratometry (SimK) values K1 and K2, which represent the flattest and the steepest respectively, and their corresponding axes, mean SimK (Average) and astigmatism values were shown in the left corners.

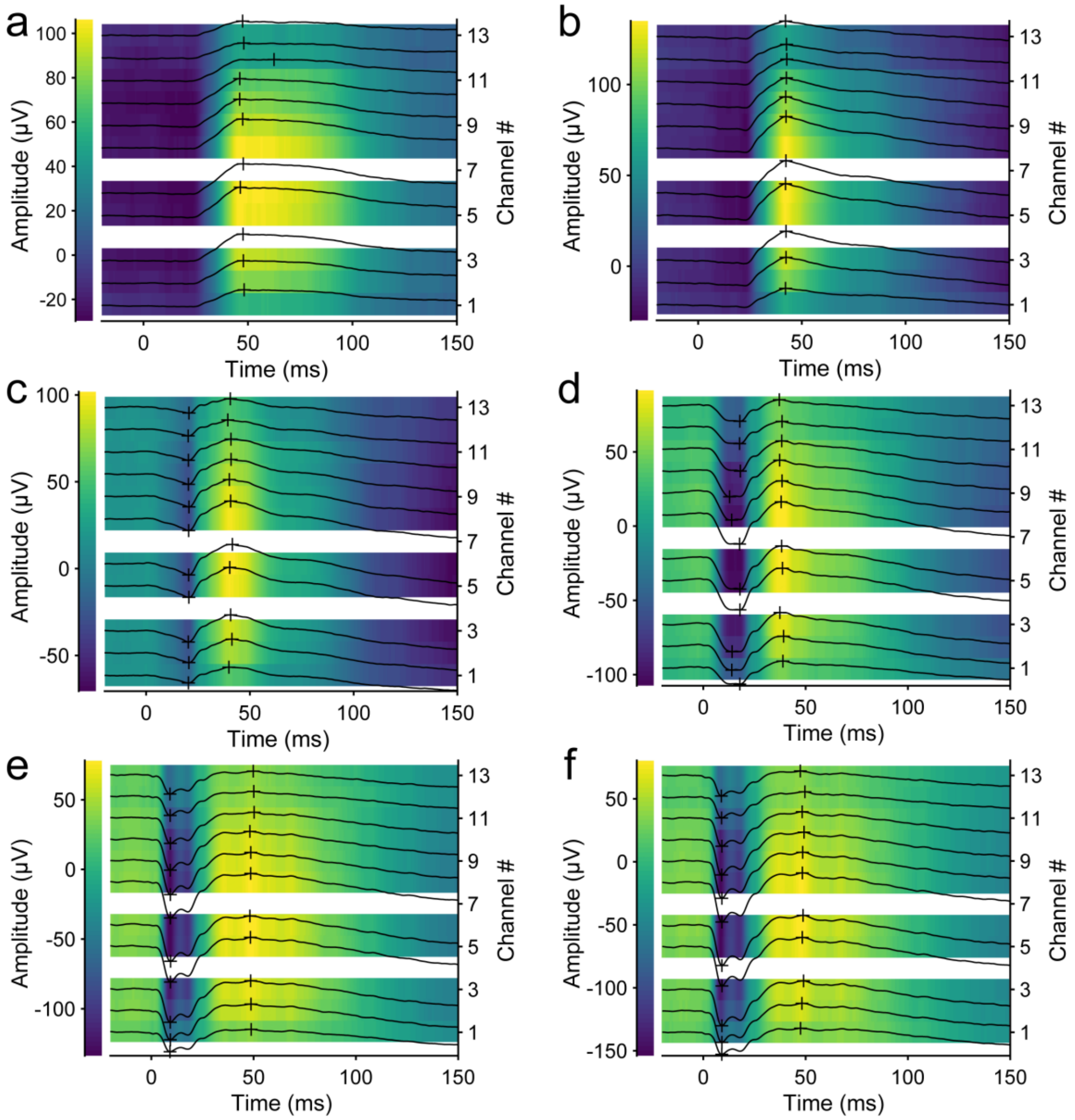

Supplementary Figure 5. Multi-electrode scotopic ERG response waveforms recorded under other stimulus intensity from the rabbit eye used in Fig. 5 *e*-*g*. *a*-*f*, 0.01, 0.03, 0.1, 1, 3.0, 10.0 cd s m^{-2}.

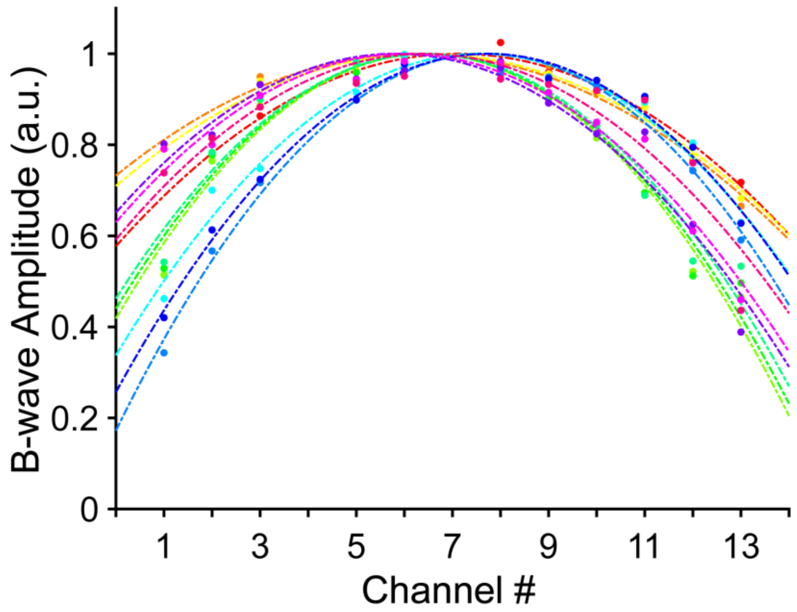

Supplementary Figure 6. Normalized b-wave amplitudes of the ERG signals recorded from different channels in meERG recordings from 12 rabbit eyes. Each color represents data from one rabbit eye. The highest potential at cornea is normalized to 1.0. The lines show the quadratic curve fitting of the normalized b-wave amplitudes.

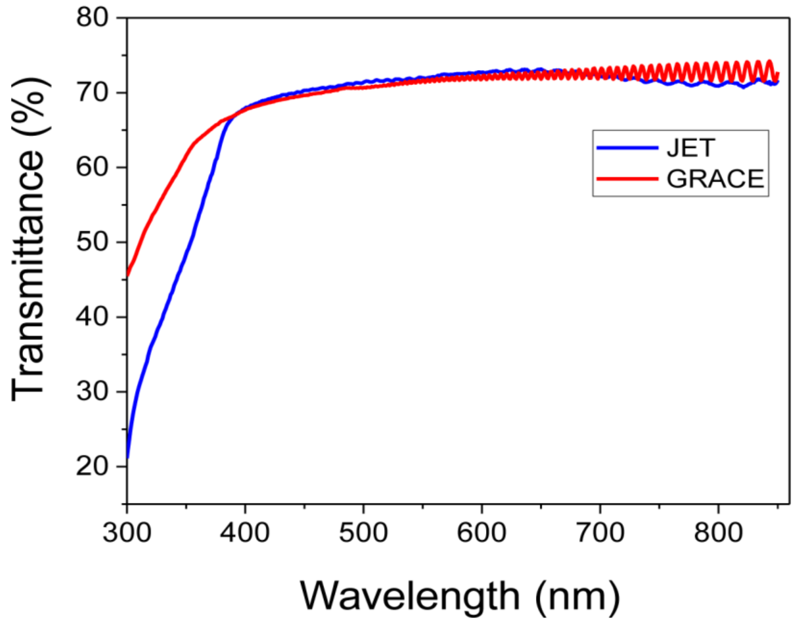

Supplementary Figure 7. Optical transmittance of the center plastic opening of a Jet electrode and a GRACE device made from G-quartz. It can be seen that these two electrodes have comparable optical transparency in the visible to near-infrared range.

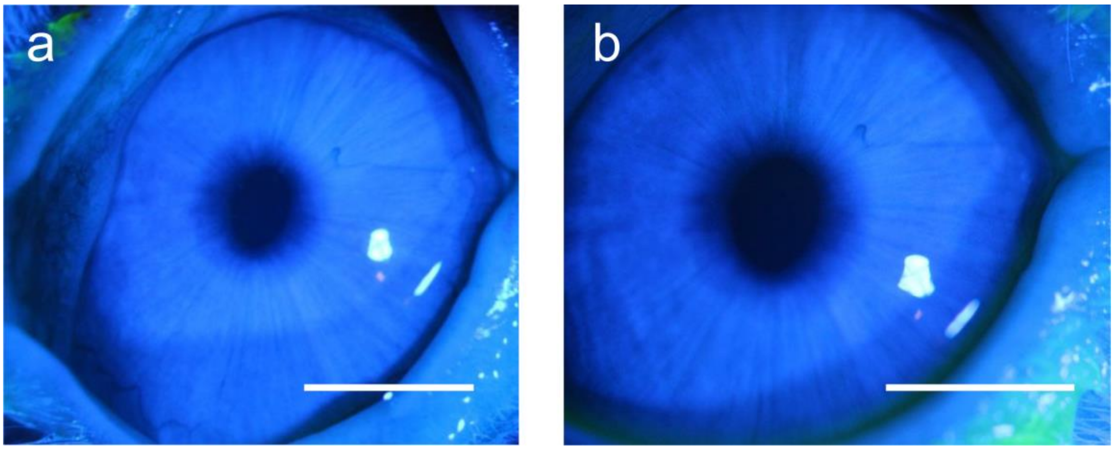

Supplementary Figure 8. Slit lamp micrographs of a rabbit eye with corneal fluorescein staining before (***a***) and after GRACE wearing for 30 min (***b***). No obvious corneal staining was observed after GRACE wearing. Scale bar, 5 mm.

Supplementary References

1 Park, A. H. *et al.* Optogenetic mapping of functional connectivity in freely moving mice via insertable wrapping electrode array beneath the skull. *ACS nano* **10**, 2791-2802 (2016).

2 Yang, X., Grosjean, C. & Tai, Y.-C. A low power MEMS silicone/parylene valve. *Electrical Engineering* **136**, 93 (1998).

3 Peng, J.-S. *et al.* Measurements of residual stresses in the Parylene C film/silicon substrate using a microcantilever beam. *J. Micromech Microeng.* **23**, 095001 (2013).

4 Keller, S. *et al.* Processing of thin SU-8 films. *J. Micromech Microeng.* **18**, 125020 (2008).
