# Supplementary material for: Soft transparent graphene contact lens electrodes for conformal full-cornea recording of electroretinogram
Source: Nat Commun. 2018 Jun 13;9:2334. doi: 10.1038/s41467-018-04781-w (PMC5998030; doi:10.1038/s41467-018-04781-w)
Supplement: Supplementary file 2 — Description of Additional Supplementary Files [file 41467_2018_4781_MOESM2_ESM.pdf]

Description of Additional Supplementary Files

File Name: Supplementary Movie 1
Description: Movie of a rabbit eye wearing a GRACE under eye blinking.

File Name: Supplementary Movie 2
Description: Movie of a rabbit with one eye wearing a GRACE showing no rubbing at eyes.
